# Pregnancy after liver transplant: maternal and perinatal outcomes

**DOI:** 10.1186/s12884-021-04104-w

**Published:** 2021-09-16

**Authors:** Izabela Marzec, Aleksandra Słowakiewicz, Jolanta Gozdowska, Olga Tronina, Marek Pacholczyk, Wojciech Lisik, Agata Fleming, Magdalena Durlik

**Affiliations:** 1 Department of Transplantation Medicine, Nephrology and Internal Medicine, Medical University of Warsaw, Nowogrodzka 59, 02-006 Warsaw, Poland; 2 Department of General and Transplantation Surgery, Medical University of Warsaw, Warsaw, Poland; 3 1st Department of Obstetrics and Gynecology, Medical University of Warsaw, Warsaw, Poland

**Keywords:** Liver transplantation, Pregnancy outcomes, Neonatal outcomes

## Abstract

**Background:**

Liver transplantation is a life-saving and successful therapeutic procedure which is more and more frequent worldwide, also among women of reproductive age. Consequently, there is an increasing number of reports of pregnancy following liver transplantation, but doubts still exist regarding preconception counseling and the optimal method of managing pregnancy. The aim of this study was to report and evaluate pregnancy outcomes in women who had undergone liver transplantation.

**Methods:**

We retrospectively analyzed female patients after orthotopic liver transplantation who reported pregnancy and were under medical care of a single transplant center.

**Results:**

We identified 14 pregnancies in 10 women who had undergone liver transplantation (12 childbirths, one induced abortion due to fetal death in the first trimester, one pregnancy is still ongoing). Causes of transplantation include congenital or acquired disorders and the most common indication was autoimmune hepatitis (50%).

The mean age at the point of transplantation was 28.5 (range 21–36), mean maternal age at pregnancy was 32 (range 26–43), and transplant-to-pregnancy interval was 4.07 years (range 1.5–7). The mean gestational week was 36.67 (range 31–40). Immunosuppression was maintained with combinations of prednisone (*n* = 11), tacrolimus (*n* = 13), and azathioprine (*n* = 8) prior to and during pregnancy. Two pregnancies were unintended, so women took mycophenolate mofetil in the first weeks of gestation. Another two women stopped taking azathioprine due to increasing anemia. Maternal complications included increase of aspartate transaminase and alanine transaminase (*n* = 2), anemia (*n* = 4) and hyperthyroidism (*n* = 2). Among the 12 childbirths, five (41.67%) were preterm. Only five women entered labor spontaneously, while seven (58,33%) had cesarean delivery.

**Conclusions:**

Pregnancy after liver transplantation can achieve relatively favorable outcomes. Liver transplantation does not influence women’s fertility and, during pregnancy, we report low rates of minor graft complications. A multidisciplinary team should be involved in contraceptive, fertility and consequently pregnancy counseling of female transplant recipients.

## Background

Liver transplantation is a treatment for acute or chronic diseases, which is an effective life-saving procedure [1–3] and is increasingly performed all over the world. The number of liver transplants is also increasing among women of childbearing (reproductive) age [3–5]. Consequently, there is an increasing number of reports of pregnancy following liver transplantation but doubts still exist regarding preconception counseling and the optimal method of managing pregnancy.

Chronic organ failure disrupts the normal functioning of the gonads, so pregnancies are relatively rare. In women, this infertility results from a changed activity of the hypothalamus, and is associated with, among others, high levels of follicle stimulating hormone (FSH) or prolactin.

In the end stage of liver failure, women complain of irregular periods or their absence, very often they are infertile [3, 6, 7]. One of the effects of successful liver transplantation is the restoration of fertility and the ability to give birth to a healthy child in up to 97% of women [3, 6, 7].

Severe irreversible organ failure is associated with metabolic and endocrine disorders, often cachexia, loss of libido. A successful organ transplant 1 year after the procedure eliminates the negative effects of the disease, including the restoration of ovulation cycles [8, 9]. There is the possibility of having biological offspring. Many patients have this additional incentive and motivation to undergo numerous medical procedures related to transplantation. However, even in 30% of women, menstruation can return immediately after transplantation [10]. Therefore, it is very important to use effective contraception in organ recipients, because a necessary condition for a trouble-free pregnancy and delivery, and for the health of the fetus and newborn is a stable function of the transplanted organ.

Natural methods of contraception are not recommended due to their low effectiveness in preventing pregnancy in women with serious underlying diseases and using immunosuppressive drugs. Women with a liver transplant who have regular intercourse should use highly effective contraceptives. The use of hormonal contraceptives is most often recommended. In the early post-transplant period, the use of hormonal contraceptives may be of concern because of the potential interaction with calcineurin inhibitors, and uncontrolled fluctuations in drug levels may cause acute organ rejection [10]. An absolute contraindication to this type of contraception is unsatisfactory function of the transplanted organ or its rejection. It is important to systematically monitor the concentration of calcineurin inhibitors.

The first pregnancy in a liver recipient was reported in 1978, 15 years after the first successful liver transplant that took place in 1963 [8, 9, 11]. The European Liver Transplant Registry reports that approximately 75% of women who have had a liver transplant are of childbearing potential. Based on this information, an increase in the number of pregnancies in liver recipients can be expected [8].

Pregnancy after organ transplantation is a significant threat to the health of the mother, child and the further functioning of the transplanted organ. The best prognosis is to plan pregnancy in women with the lowest risk of rejection, while maintenance doses of immunosuppressants are used.

## Methods

The aim of this study is to report and evaluate pregnancy outcomes in women who underwent liver transplantation. We retrospectively analyzed medical records of 10 women who had undergone liver transplantation at the Department of General and Transplantation Surgery and were under further care of the doctors of the Department of Transplantation Medicine, Nephrology and Internal Medicine. The pregnancies were supervised at the Gynecology and Obstetrics Clinic of the Medical University of Warsaw. We enrolled only those patients about whom full data were obtained. We extracted data on: details of the liver transplantation and graft health before and after pregnancy, immunosuppressive therapy and liver function, comorbidities, pregnancy-related outcomes (such as mode of conception and number of fetuses), maternal complications such as maternal death, graft rejection or failure, way of ending pregnancy, date of delivery: weight, length and number of points on the APGAR scale of the child after delivery. We also presented data on long-term follow-up of both the mother (medical records) and the infant (data on children obtained from mothers).

## Results

### Maternal characteristics

We identified 14 pregnancies in 10 women who underwent liver transplantation - 12 births and one abortion-induced death during the first trimester of pregnancy. The fourteenth pregnancy was ongoing, the patient was in the third trimester of pregnancy, the pregnancy was uneventful. Due to incomplete data, we did not include this pregnancy in the statistics.

The mean age at the point of transplantation was 28.5 (range 21–36), mean maternal age at pregnancy was 32 (range 26–43), and transplant-to-pregnancy interval was 4.07 years (range 1.5–7). Causes of transplant include congenital or acquired disorders described in Table 1.

**Table 1:** Indications for liver transplantation

Indication	Number of patients
Autoimmune hepatitis (AIH)	5
Wilson’s disease	3
Budd–Chiari syndrome	2
HCV	1

The most common indication for liver transplantation was autoimmune hepatitis (50%). The patient with HCV had no active infection at the time of pregnancy (after therapy DAA, HCV RNA tested by PCR was negative).

Immunosuppression was maintained with combinations of prednisone (*n* = 11), tacrolimus (*n* = 13), and azathioprine (*n* = 8) prior to and during pregnancy. Two pregnancies were unintended, so women took mycophenolate mofetil in the first weeks of gestation. Another two women stopped taking azathioprine due to increasing anemia.

Maternal complications included increase of aspartate transaminase and alanine transaminase (*n* = 2), anemia (*n* = 4), hyperthyroidism (*n* = 2), leukopenia and thrombocytopenia (*n* = 1), asymptomatic leukocyturia (*n* = 1) and erythrocyturia (*n* = 1). One of the patients with hyperthyroidism had AIH and the other Wilson’s disease – it is rather a coincidence.

One patient was found to have an active HBV infection during pregnancy. In accordance with the guidelines, treatment with tenofovir was started in the third trimester (the only drug approved for the treatment of pregnant women). The child received anti-HBs globulin, was vaccinated on the first day and has no HBV DNA.

Laboratory results are presented in Table 2.

**Table 2:** Mean laboratory results

	PRE-PREGNANCY	PRE-LABOUR	POST-LABOUR	P
WBC (G/l)	5.91	7.40	5.35	NS
RBC (T/l)	4.63	3.57	3.74	< 0.05
HGB (mg/dl)	13.03	11.17	11.51	< 0.05
PLT (G/l)	209.62	159.25	198.25	NS
Glucose (mg/dl)	82.08	82.00	83.18	NS
ALT (U/l)	30.69	30.08	22.83	NS
AST (U/l)	25.92	25.58	19.25	NS
Bilirubin (mg/dl)	0.55	0.50	0.47	NS
GGT (U/l)	21.23	12.10	20.50	NS
Albumin (g/dl)	4.41	3.22	3.92	NS
INR	1.2	1.1	1.2	NS
Tacrolimus (ng/ml)	5.9	5.1	5.3	NS

### Pregnancy outcomes

There were no maternal deaths. The general gestational age was 36.67 (range 31–40). Among the 12 total childbirths, five (41.67%) delivered preterm, with a range between 31 and 36 weeks of gestation. Only five women entered labor spontaneously, while seven (58.33%) were delivered by caesarean section. Analyzing a subgroup of seven patients that delivered at term revealed a high rate of cesarean (four cases, 57.14%) with obstetric indications. In the subgroup of premature deliveries (five cases), the percentage of cesarean was similar (60%). The degree of anemia had no effect on preterm labor.

Only one woman breastfed her child. It was the patient’s choice after consultation with gynecologists.

### Neonatal outcomes

There were seven female and five male newborns. The average weight at birth was 2775.83 g (range 1140–3600 g) and the average length was 51.33 cm (range 38–58 cm). Five of 12 neonates (41.67%) demonstrated low birth weights, which were defined as less than 2.500 g. The lowest birth weight occurred in a newborn weighing 1140 g, who was born in 31 Hbd and diagnosed with intrauterine hypotrophy. Clinical characteristics at birth were stable, with an Apgar score > 8 in almost all newborns. Three infants required assisted breathing (CPAP – Continuous Positive Airway Pressure), with a subsequent progressive improvement of the clinical conditions. There were no neonatal deaths.

### Children outcomes

In our study, we assessed the long-term development of 12 children. The mean age of the children was 6.08 years (range 1–14 years). All schoolchildren are harmoniously developed, both psychomotor and psychologically (without intellectual disabilities) and do not differ from their peers.

One child was diagnosed with attention deficit hyperactivity disorder (ADHD). One child was born with one healthy kidney. One child had patent ductus arteriosus. One child was diagnosed with mild tricuspid regurgitation. Both children are under constant supervision of a cardiologist and their development is normal.

### Maternal long-term outcomes

The mean follow-up of 10 women who underwent liver transplantation was 5.3 years (range 1–11 years). We found no cases of graft loss. One patient (2 years after the second pregnancy) developed acute liver rejection and concomitant AIH. She was treated with pulses of methylprednisolone and is currently receiving steroids, MMF and tacrolimus. She is a non-adherent patient. Another patient with a history of thrombophilia experienced visual disturbances and dural sinus thrombosis and right internal jugular vein thrombosis. In addition, this patient had a Tac / CsA conversion due to suspected tacrolimus toxicity. One patient experienced exacerbation of ulcerative colitis. Another patient experienced transient abdominal pain and suspected IBD. One patient developed cirrhosis of the liver (abnormal liver function tests - LFTs). The remaining nine patients had normal liver function. The mean results of laboratory tests both before pregnancy and in long-term follow-up are presented in Table 3.

**Table 3:** Mean laboratory results

	PRE-PREGNANCY	LONG-TERM OUTCOME	P
WBC (G/l)	5.91	6.55	NS
RBC (T/l)	4.63	4.72	NS
HGB (mg/dl)	13.03	13.36	NS
PLT (G/l)	209.62	226.5	NS
Glukoza (mg/dl)	82.08	86.6	NS
ALAT (U/l)	30.69	20.6	NS
ASPAT (U/l)	25.92	21	NS
Bilirubina (mg/dl)	0.55	0.72	NS
GGTP (U/l)	21.23	73.7	NS
Albuminy (g/dl)	4.41	4.33	NS
INR	1.2	1.49	NS
Takrolimus (ng/ml)	5.9	5.88	NS

## Discussion

Women with chronic liver disease have a very low chance of conceiving and carrying a pregnancy. It is caused by irregular menstrual cycles and often no menstruation at all. Liver transplantation gives women a chance to become mothers. It was noticed that even 6 weeks after transplantation, female fertility returns. Almost 90% of female recipients have regular periods 1 year after the liver transplant [8, 12].

The latest research shows that with the proper functioning of the transplant, a woman can give birth to a healthy child. It is recommended that a woman should wait at least 1 year (preferably 2 years) after the transplant to begin trying to get pregnant. If conception occurs in the first year after transplantation, there is a high risk of prematurity and graft rejection. In our study, the mean transplant-to-pregnancy interval was 4.07 years.

In immunosuppressive treatment regimens, several drugs with different mechanisms of action (usually 2 or 3) are combined. These drugs include: calcineurin inhibitors (tacrolimus, tacrolimus with modified release, tacrolimus with extended release (LCPT)), mycophenolates (mycophenolate sodium, mycophenolate mofetil), glucocorticosteroids, drugs inhibiting cell division (azathioprine) and mTOR inhibitors (sirolimus). The most common regimen used in the treatment of a vascularized organ transplant patient is calcineurin inhibitor + mycophenolate + glucocorticosteroid [13].

Pregnancy is not a reason to discontinue immunosuppressive therapy. Organ recipients must take immunosuppressants throughout pregnancy to prevent organ rejection and loss. A patient expecting a baby can safely use drugs such as glucocorticosteroids, tacrolimus or cyclosporine A and azathioprine. However, mycophenolates and mTOR inhibitors are contraindicated [12, 14].

In our center, if at least 6 weeks before a planned pregnancy, immunosuppression is modified. Mycophenolate mofetyl is discontinued and azathioprine is included.

Analysis of the concentration of immunosuppressive drugs should be prescribed by a transplant physician in order to correct the dose every 4–8 weeks. An increase of serum volume in pregnancy can lead to a decrease of drug concentrations. On the contrary, excess storage in the third space and adipose tissue is also possible. Therefore, we recommend that immunosuppressant concentrations are assessed every 4 weeks and that the doses are adjusted accordingly.

A wide range of graft rejection prevalence has been reported in many studies, ranging from 0 to 17% [6, 14–16]. In our study, patients were not diagnosed with acute rejection episodes (0%) during pregnancy.

There is more and more data worldwide of pregnant transplant recipients, and the course of pregnancy and its complications is the subject of many studies, which allows specialized multidisciplinary teams to take optimal care for this high-risk patients group. Fetal development should be monitored with equal care, as well as the mother’s health and the function of the transplanted organ.

Women who have had a liver transplant during pregnancy and puerperium are at risk of many complications. Pregnancy complications include: anemia, gestational diabetes, hypertension and pre-eclampsia, and gestational cholestasis. The most common complications observed during pregnancy include increased levels of aspartate and alanine transaminase, as well as anemia and urinary tract infection [8, 17]. Peripartum complications are preterm labor, haemorrhage, the need for blood transfusions [8].

Anemia is associated with both the long-term use of immunosuppressive drugs and the physiology of pregnancy itself [8]. In our study, 40% of the women surveyed suffered from anemia during pregnancy. Blood transfusions were required in three cases. The values of hemoglobin and erythrocytes in laboratory results were statistically significantly decreased (*p* < 0.05).

In two women (20%) an increased activity of aspartate transaminase and alanine transaminase was observed; hyperthyroidism was also observed in 20% of women; likewise, leukopenia and thrombocytopenia were observed in 20% of women, while 10% suffered from asymptomatic leukocyturia and erythrocyturia.

Additional risk factors for the course of pregnancy may include the mother’s age, her medical history with multiple surgical interventions, and the mother’s underlying disease.

The next stage of our work was the analysis of the results for fetuses and newborns born to women after liver transplantation. According to The US National Transplantation Pregnancy Registry (NTPR) in 2017 between 84,6% and 71,9% of pregnancies in women after liver transplantation ended with the birth of a live child [12]. In our study, the percentage of live births was as high as 92.3%. There was only one spontaneous miscarriage in the first trimester of pregnancy, and no stillbirths.

Many authors mention prematurity and inhibition of intrauterine growth as the most common complications [8, 14, 18]. In the literature, the prevalence of prematurity varies between 27 and 30%, and the incidence of intrauterine growth inhibition is 5–20%. In our study group, 41.67% of children were born prematurely, and 8.33% (*n* = 1) had intrauterine hypotrophy.

The conducted study was limited by a small number of patients (*n* = 10) and only 12 pregnancies, that were analysed but in the group subjected to detailed analysis, we did not observe an increased risk of birth defects in the fetus. The results of our study confirm that the immunosuppressants used (prednisone, tacrolimus, azathioprine) do not adversely affect the fetus.

Due to postoperative wounds and adhesions, vaginal delivery is preferred in women after transplantation. However, there is now an increasing trend towards cesarean delivery, which is also reflected in transplant patients. All cesarean sections in our study group accounted for as much as 58.33% of all deliveries, and all of them had obstetric indications. The c-section rate in Poland reaches 44% in the general population (the European average of around 27%). After liver transplantation pooled rates of delivery outcomes for caesarean section (C-section) and pre-term birth were 42.2 and 27.8% [19].

Breastfeeding for transplant recipient mothers remains an area of uncertainty. In 2014, the Transplantation Pregnancy Registry published a review of breastfeeding after transplantation and concluded from the literature and registry data that it appears to be safe to breastfeed while taking normal maintenance doses of prednisone, azathioprine, cyclosporine and tacrolimus [20]. While any immunosuppressive drug exposure to the infant could potentially exceed the threshold for safety, the relatively small amount of drug transferred through breast milk and the lack of reported adverse effects together with the documented benefits of breastfeeding may outweigh the theoretical risks of this exposure. Testing of the level of immunosuppressive drugs in the blood of the child shortly after birth should allay fears, because they quickly become immeasurable. Breastfeeding is inadvisable while taking MMF, sirolimus, everolimus and belatacept based on theoretical and pharmacologic concerns [12, 18].

Fetal development should be monitored with equal care as well as the mother’s health and the function of the transplanted organ. Also of interest is the fact that the our patents’ children develop properly after several years of observation. They do not stand out from their peers both in terms of growth and intellectual development.

## Conclusions

In conclusion, pregnancies after liver transplantation have a good chance of being successful. Nevertheless, pregnancy in female recipients is much riskier than in the general population of healthy women. Therefore, the issue of fertility and maternity planning should be widely discussed with the patient and her partner. A pregnant patient and her child should be looked after by a multidisciplinary team to ensure their safety as much as possible. The long-term health status of liver transplant recipients and their babies should continue to be assessed.

## Data Availability

The datasets used and analysed during the current study available from the corresponding author on reasonable request.

## Abbreviations

FSH: follicle stimulating hormone
MMF: mycophenolate mofetil
AIH: autoimmune hepatitis
CPAP: Continuous Positive Airway Pressure
ADHD: attention-deficit hyperactivity disorder
NTPR: National Transplantation Pregnancy Registry
LCPT: tacrolimus with extended release
